# Disodium tricopper(II) tetra­kis[selenate(IV)] tetra­hydrate

**DOI:** 10.1107/S160053680803434X

**Published:** 2008-10-25

**Authors:** Wei Liu, Lan Zhang, Kai Hu, Lixin Cao, Ge Su, Jingtai Zhao

**Affiliations:** a Institute of Materials Science and Engineering, The Ocean University of China, Qingdao 266100, People’s Republic of China; b Department of Computer Science, Dezhou University, Shandong 253023, People’s Republic of China; c State Key Laboratory of High Performance Ceramics and Superfine Microstructure, Shanghai Institute of Ceramics, Chinese Academy of Science, Shanghai 200050, People’s Republic of China

## Abstract

The title compound, Na_2_Cu_3_(SeO_3_)_4_(H_2_O)_4_, has been prepared under hydro­thermal conditions. The crystal structure contains a three-dimensional anionic framework made up from distorted [CuO_4_(H_2_O)_2_] octa­hedra (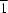 symmetry), [CuO_4_(H_2_O)] square pyramids and trigonal-pyramidal SeO_3_ units sharing common corners. The connectivity among these units leads to four- and eight-membered polyhedral rings, which by edge-sharing inter­connect into walls. A rhombus-like 16-membered polyhedral ring channel system with a longest length of approximately 14.0 Å and a shortest length of 5.3 Å is enclosed by such walls along the *a* axis. The water mol­ecules attached to the Cu atoms, as well as the electron lone pairs of the Se^IV^ atoms, protrude into these channels. The seven-coordinated Na^+^ counter-cations occupy the remaining free space of the 16-membered polyhedral ring channels. An intricate network of O—H⋯O hydrogen bonds further consolidates the three-dimensional structure.

## Related literature

For the structures of other hydrous copper(II) selenates(IV) or selenates(VI), see: Asai & Kiriyama (1973 ▶), Giester (1991 ▶); Iskhakova & Kozlova (1995 ▶).

## Experimental

### 

#### Crystal data

Na_2_Cu_3_(SeO_3_)_4_(H_2_O)_4_
*M* *_r_* = 816.50
Monoclinic, 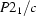

*a* = 5.2218 (5) Å
*b* = 8.9863 (6) Å
*c* = 15.7960 (11) Å
β = 92.071 (4)°
*V* = 740.74 (10) Å^3^
*Z* = 2
Mo *K*α radiation
μ = 14.24 mm^−1^
*T* = 296 (2) K
0.15 × 0.10 × 0.10 mm

#### Data collection

Bruker SMART CCD diffractometer
Absorption correction: multi-scan (*SADABS*; Sheldrick, 2004 ▶) *T*_min_ = 0.204, *T*_max_ = 0.250
6010 measured reflections
2142 independent reflections
2065 reflections with *I* > 2σ(*I*)
*R*_int_ = 0.027

#### Refinement

*R*[*F* ^2^ > 2σ(*F* ^2^)] = 0.033
*wR*(*F* ^2^) = 0.068
*S* = 1.12
2142 reflections
115 parameters
H atoms treated by a mixture of independent and constrained refinement
Δρ_max_ = 0.77 e Å^−3^
Δρ_min_ = −1.02 e Å^−3^

### 

Data collection: *SMART* (Bruker, 2001 ▶); cell refinement: *SAINT* (Bruker, 2001 ▶); data reduction: *SAINT*; program(s) used to solve structure: *SHELXS97* (Sheldrick, 2008 ▶); program(s) used to refine structure: *SHELXL97* (Sheldrick, 2008 ▶); molecular graphics: *ORTEP-3* (Farrugia, 1997 ▶) and *DIAMOND* (Crystal Impact, 2004 ▶); software used to prepare material for publication: *WinGX* (Farrugia, 1999 ▶).

## Supplementary Material

Crystal structure: contains datablocks I, global. DOI: 10.1107/S160053680803434X/wm2197sup1.cif

Structure factors: contains datablocks I. DOI: 10.1107/S160053680803434X/wm2197Isup2.hkl

Additional supplementary materials:  crystallographic information; 3D view; checkCIF report

## Figures and Tables

**Table 1:** Selected bond lengths (Å)

Se1—O7	1.698 (3)
Se1—O2	1.705 (3)
Se1—O4	1.709 (3)
Se2—O8	1.673 (3)
Se2—O1	1.708 (3)
Se2—O3	1.717 (3)
Cu1—O4	1.968 (3)
Cu1—O6	1.990 (3)
Cu1—O8^i^	2.475 (3)
Cu2—O1^ii^	1.947 (3)
Cu2—O3	1.962 (3)
Cu2—O2	1.968 (3)
Cu2—O7^iii^	1.980 (3)
Cu2—O5	2.268 (3)

Symmetry codes: (i) 
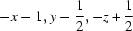
; (ii) 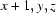; (iii) 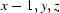.

**Table 2:** Hydrogen-bond geometry (Å, °)

*D*—H⋯*A*	*D*—H	H⋯*A*	*D*⋯*A*	*D*—H⋯*A*
O5—H1⋯O4^iv^	0.80 (8)	2.00 (8)	2.786 (4)	168 (7)
O5—H2⋯O3^v^	0.87 (8)	1.88 (8)	2.746 (4)	174 (7)
O6—H3⋯O8^vi^	0.87 (8)	1.91 (8)	2.758 (5)	163 (7)
O6—H4⋯O1^i^	0.89 (8)	1.76 (8)	2.641 (4)	169 (8)

Symmetry codes: (i) 
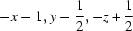
; (iv) 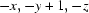; (v) 
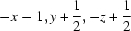
; (vi) 
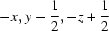
.

**Fig. 1.**
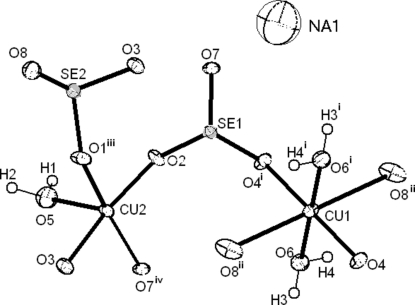
The coordination environment of copper and selenium atoms with anisotropic thermal ellipsoids drawn at the 60% probability level. H atoms are draw as small spheres of arbitrary radius. [Symmetry codes: (i) -x, -y, -z; (ii) -x - 1, y - 1/2, -z + 1/2; (iii) x + 1, y, z; (iv) x - 1, y, z).]

**Fig. 2.**
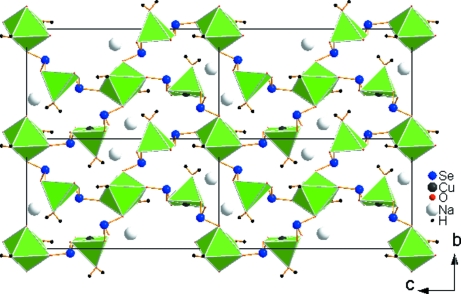
The 16-membered polyhedral ring channels of (I), filled with Na+ counter cations.

**Fig. 3.**
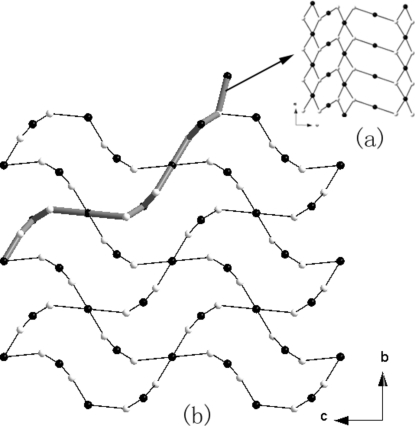
Schematic representation of the formation of the anionic framework structure of (I). (a): The wavy layer built from an edge-sharing linear chain by corner-sharing 4-membered rings and ladders by edge-sharing 8-membered rings; (b): The wavy layers intersecting each other with an approximate angle of 110° to the entire three-dimensional open-framework. (Black spheres: Cu atoms; white spheres: Se atoms).

## supplementary crystallographic information

### Comment

Studies of hydrous copper selenites and selenates with three-dimensional frameworks have been reported previously, e.g. by Asai & Kiriyama (1973), Giester (1991) and Iskhakova & Kozlova (1995). Among the corresponding structures various polyhedral ring channel systems are established. The current article presents the result of the single-crystal X-ray analysis of a new sodium copper selenite, Na_2_Cu_3_(SeO_3_)_4_(H_2_O)_4_, (I), with a 16-membered polydedral ring channel system.

In the asymmetric unit of (I) there are two crystallographically distinct copper atoms. The six-coordinated Cu1 site is a typical Jahn-Teller ion with a distorted, tetragonally elongated octahedral [Cu1O_4_(H_2_O)_2_] coordination, whereas Cu2 is surrounded by five O-atoms, leading to a distorted square-pyramidal [Cu2O_4_(H_2_O)] environment. The two independent selenium atoms are coordinated by three oxygen atoms, forming the characteristic trigonal-pyramidal SeO_3_^2-^ anion (Fig. 1).

The square-pyramidal [Cu2O_4_(H_2_O)] units share its basal O atoms with four neighboring SeO_3_ units leading to chains of corner-shared four-membered polyhedral rings running along [100]. The [Cu1O_4_(H_2_O)_2_] units are located between such parallel chains and bridge them *via* Cu—O—Se bonds into an open framework. The water molecules attached to Cu1 and Cu2 as well as the electron lone-pairs of the selenium(IV) atoms protrude into the free space of this network (Fig. 2).

The basic features of the structure could also be described as the assemblage of linear chains of Cu and Se centres leading to 4-membered and 8-membered rings that interconnect by edge-sharing into two similar wavy layer packings extending along [011] and [011], respectively. Such layers intersect at the Cu(1) sites, eventually forming a rhombus-like 16-membered ring channel system extending along the *a* axis with the biggest length of approximately 14.0 Å and the smallest length of 5.3 Å (Fig.3).

The Na^+^ counter cations are coordinated by seven oxygen atoms and occupy the central space of the 16-membered ring polyhedral channels to keep the structural stability and satisfy the charge balance. An intricate network of O—H···O hydrogen bonds further consolidates the three-dimensional structure (Table 2).

### Experimental

The title compound was synthesized under hydrothermal conditions. A mixture of Na_2_SeO_3_ and CuSO_4_^.^5H_2_O in a molar ratio of 1:1 was placed in a Teflon-lined stainless steel autoclave and heated to 443 K for 5 d, cooled at 2 K/h to 373 K, and finally cooled to room temperature. Light blue crystals with a rod-like habit were obtained. Cu, Se and Na contents were analyzed using ICP-AES (Varian Vista, radial observation): Obs./calc. mass%: Cu, 23.33/23.91; Se, 38.70/39.23; Na, 5.63/5.42.

### Refinement

Charge balance considerations and bond valence sum (BVS) calculations indicate that atoms O5 and O6 belong to water molecules. For the metal atoms, the oxidation states of +2 for Cu ions (BVS Cu1: +2.05 and Cu2: +2.01), +4 for the Se ions (BVS Se1: +4.01 and Se2: +4.06) and +1 for the Na ions (BVS Na1: +1.02) were confirmed. The hydrogen atoms of the water molecules were located from difference Fourier maps and were refined with distance restraints of d(O—H) = 0.80 (8)–0.89 (8) Å and a common U_iso_ parameter of 0.05 Å^2^.

### Crystal data

**Table:**

Na_2_Cu_3_(SeO_3_)_4_(H_2_O)_4_	*F*(000) = 762
*M**_r_* = 816.50	*D*_x_ = 3.661 Mg m^−^^3^
Monoclinic, *P*2_1_/*c*	Mo *K*α radiation, λ = 0.71073 Å
Hall symbol: -P 2ybc	Cell parameters from 986 reflections
*a* = 5.2218 (5) Å	θ = 2.6–22.8°
*b* = 8.9863 (6) Å	µ = 14.24 mm^−^^1^
*c* = 15.7960 (11) Å	*T* = 296 K
β = 92.071 (4)°	Rod, light blue
*V* = 740.74 (10) Å^3^	0.15 × 0.10 × 0.10 mm
*Z* = 2	

### Data collection

**Table:**

Bruker SMART CCD diffractometer	2142 independent reflections
Radiation source: fine-focus sealed tube	2065 reflections with *I* > 2σ(*I*)
graphite	*R*_int_ = 0.027
ω scans	θ_max_ = 30.0°, θ_min_ = 2.6°
Absorption correction: multi-scan (SADABS; Sheldrick, 2004)	*h* = −7→7
*T*_min_ = 0.204, *T*_max_ = 0.250	*k* = −12→12
6010 measured reflections	*l* = −21→22

### Refinement

**Table:**

Refinement on *F*^2^	Primary atom site location: structure-invariant direct methods
Least-squares matrix: full	Secondary atom site location: difference Fourier map
*R*[*F*^2^ > 2σ(*F*^2^)] = 0.033	Hydrogen site location: inferred from neighbouring sites
*wR*(*F*^2^) = 0.068	H atoms treated by a mixture of independent and constrained refinement
*S* = 1.12	*w* = 1/[σ^2^(*F*_o_^2^) + (0.0202*P*)^2^ + 3.7977*P*] where *P* = (*F*_o_^2^ + 2*F*_c_^2^)/3
2142 reflections	(Δ/σ)_max_ = 0.001
115 parameters	Δρ_max_ = 0.77 e Å^−^^3^
0 restraints	Δρ_min_ = −1.02 e Å^−^^3^

### Special details

**Table:**

Geometry. All e.s.d.'s (except the e.s.d. in the dihedral angle between two l.s. planes) are estimated using the full covariance matrix. The cell e.s.d.'s are taken into account individually in the estimation of e.s.d.'s in distances, angles and torsion angles; correlations between e.s.d.'s in cell parameters are only used when they are defined by crystal symmetry. An approximate (isotropic) treatment of cell e.s.d.'s is used for estimating e.s.d.'s involving l.s. planes.
Refinement. Refinement of *F*^2^ against ALL reflections. The weighted *R*-factor *wR* and goodness of fit *S* are based on *F*^2^, conventional *R*-factors *R* are based on *F*, with *F* set to zero for negative *F*^2^. The threshold expression of *F*^2^ > σ(*F*^2^) is used only for calculating *R*-factors(gt) *etc*. and is not relevant to the choice of reflections for refinement. *R*-factors based on *F*^2^ are statistically about twice as large as those based on *F*, and *R*- factors based on ALL data will be even larger.

### Fractional atomic coordinates and isotropic or equivalent isotropic displacement parameters (Å^2^)

**Table:**

	*x*	*y*	*z*	*U*_iso_*/*U*_eq_	
Se1	0.20254 (7)	0.28287 (4)	0.09687 (2)	0.01090 (10)	
Se2	−0.79415 (7)	0.53958 (5)	0.27304 (2)	0.01476 (11)	
Cu1	0.0000	0.0000	0.0000	0.01381 (14)	
Cu2	−0.29891 (8)	0.42910 (6)	0.17116 (3)	0.01266 (11)	
O1	−1.0733 (5)	0.4403 (4)	0.27181 (18)	0.0207 (6)	
O2	−0.0088 (5)	0.4276 (3)	0.09497 (18)	0.0153 (5)	
O3	−0.5831 (5)	0.4016 (3)	0.24757 (18)	0.0165 (5)	
O4	0.1289 (6)	0.2058 (3)	0.00011 (18)	0.0166 (5)	
O5	−0.3653 (7)	0.6725 (4)	0.1392 (2)	0.0209 (6)	
O6	0.2781 (6)	−0.0756 (4)	0.07804 (19)	0.0199 (6)	
O7	0.4702 (5)	0.3793 (3)	0.07309 (17)	0.0149 (5)	
O8	−0.7371 (6)	0.5600 (4)	0.3773 (2)	0.0272 (7)	
Na1	0.7415 (3)	0.3619 (2)	−0.04070 (11)	0.0226 (4)	
H1	−0.277 (15)	0.707 (9)	0.104 (5)	0.050*	
H2	−0.372 (14)	0.748 (9)	0.174 (5)	0.050*	
H4	0.226 (15)	−0.063 (9)	0.131 (5)	0.050*	
H3	0.437 (15)	−0.049 (9)	0.088 (5)	0.050*	

### Atomic displacement parameters (Å^2^)

**Table:**

	*U*^11^	*U*^22^	*U*^33^	*U*^12^	*U*^13^	*U*^23^
Se1	0.01040 (17)	0.01286 (18)	0.00949 (17)	−0.00189 (12)	0.00133 (12)	−0.00064 (12)
Se2	0.01172 (18)	0.0176 (2)	0.01500 (18)	0.00083 (13)	0.00180 (13)	−0.00071 (14)
Cu1	0.0139 (3)	0.0124 (3)	0.0149 (3)	−0.0023 (2)	−0.0030 (2)	−0.0004 (2)
Cu2	0.0085 (2)	0.0195 (2)	0.0101 (2)	0.00022 (17)	0.00143 (15)	−0.00087 (17)
O1	0.0097 (12)	0.0415 (19)	0.0110 (12)	−0.0042 (12)	0.0007 (10)	−0.0034 (12)
O2	0.0107 (12)	0.0208 (14)	0.0146 (12)	0.0036 (10)	0.0042 (10)	0.0019 (11)
O3	0.0137 (12)	0.0207 (14)	0.0152 (12)	0.0002 (11)	0.0044 (10)	0.0009 (11)
O4	0.0211 (14)	0.0166 (13)	0.0121 (12)	−0.0080 (11)	0.0015 (10)	−0.0022 (10)
O5	0.0298 (16)	0.0117 (14)	0.0215 (15)	−0.0011 (12)	0.0048 (13)	−0.0013 (11)
O6	0.0177 (14)	0.0280 (17)	0.0138 (13)	0.0011 (12)	−0.0030 (11)	−0.0043 (12)
O7	0.0099 (11)	0.0215 (14)	0.0135 (12)	−0.0054 (10)	0.0010 (9)	−0.0021 (11)
O8	0.0190 (14)	0.045 (2)	0.0176 (14)	−0.0028 (14)	0.0012 (12)	−0.0149 (14)
Na1	0.0193 (8)	0.0307 (10)	0.0177 (8)	0.0015 (7)	0.0015 (6)	0.0005 (7)

### Geometric parameters (Å, °)

**Table:**

Se1—O7	1.698 (3)	Cu2—O1^iii^	1.947 (3)
Se1—O2	1.705 (3)	Cu2—O3	1.962 (3)
Se1—O4	1.709 (3)	Cu2—O2	1.968 (3)
Se2—O8	1.673 (3)	Cu2—O7^iv^	1.980 (3)
Se2—O1	1.708 (3)	Cu2—O5	2.268 (3)
Se2—O3	1.717 (3)	Na1—O7	2.333 (4)
Cu1—O4	1.968 (3)	Na1—O5^v^	2.482 (4)
Cu1—O4^i^	1.968 (3)	Na1—O2^vi^	2.519 (4)
Cu1—O6	1.990 (3)	Na1—O4^iii^	2.526 (4)
Cu1—O6^i^	1.990 (3)	Na1—O2^iii^	2.537 (3)
Cu1—O8^ii^	2.475 (3)	Na1—O7^vi^	2.618 (4)
Cu1—O8^ii^	2.475 (3)	Na1—O6^vii^	2.641 (4)
			
O7—Se1—O2	98.30 (14)	Cu2—O5—H2	129 (5)
O7—Se1—O4	99.75 (13)	Na1^v^—O5—H2	116 (5)
O2—Se1—O4	99.71 (14)	H1—O5—H2	100 (7)
O8—Se2—O1	100.95 (16)	Cu1—O6—Na1^vii^	99.94 (13)
O8—Se2—O3	102.54 (16)	Cu1—O6—H4	107 (5)
O1—Se2—O3	100.08 (15)	Na1^vii^—O6—H4	109 (5)
O4—Cu1—O4^i^	180.00 (6)	Cu1—O6—H3	133 (5)
O4—Cu1—O6	94.50 (13)	Na1^vii^—O6—H3	110 (5)
O4^i^—Cu1—O6	85.50 (13)	H4—O6—H3	97 (7)
O4—Cu1—O6^i^	85.50 (13)	Se1—O7—Cu2^iii^	115.16 (15)
O4^i^—Cu1—O6^i^	94.50 (13)	Se1—O7—Na1	131.60 (16)
O6—Cu1—O6^i^	180.00 (14)	Cu2^iii^—O7—Na1	104.37 (12)
O1^iii^—Cu2—O3	87.30 (12)	Se1—O7—Na1^vi^	98.71 (13)
O1^iii^—Cu2—O2	92.48 (12)	Cu2^iii^—O7—Na1^vi^	101.13 (12)
O3—Cu2—O2	172.34 (13)	Na1—O7—Na1^vi^	99.95 (12)
O1^iii^—Cu2—O7^iv^	169.80 (14)	O7—Na1—O5^v^	90.14 (12)
O3—Cu2—O7^iv^	90.00 (12)	O7—Na1—O2^vi^	124.83 (13)
O2—Cu2—O7^iv^	88.88 (11)	O5^v^—Na1—O2^vi^	108.41 (12)
O1^iii^—Cu2—O5	102.39 (14)	O7—Na1—O4^iii^	110.09 (12)
O3—Cu2—O5	98.36 (13)	O5^v^—Na1—O4^iii^	134.38 (13)
O2—Cu2—O5	89.17 (12)	O2^vi^—Na1—O4^iii^	93.18 (11)
O7^iv^—Cu2—O5	87.73 (12)	O7—Na1—O2^iii^	69.01 (10)
Se2—O1—Cu2^iv^	121.82 (17)	O5^v^—Na1—O2^iii^	158.39 (13)
Se1—O2—Cu2	120.42 (16)	O2^vi^—Na1—O2^iii^	80.72 (11)
Se1—O2—Na1^vi^	102.26 (13)	O4^iii^—Na1—O2^iii^	62.06 (10)
Cu2—O2—Na1^vi^	130.61 (15)	O7—Na1—O7^vi^	80.06 (12)
Se1—O2—Na1^iv^	98.62 (14)	O5^v^—Na1—O7^vi^	70.66 (11)
Cu2—O2—Na1^iv^	97.75 (12)	O2^vi^—Na1—O7^vi^	60.11 (10)
Na1^vi^—O2—Na1^iv^	99.28 (11)	O4^iii^—Na1—O7^vi^	150.69 (12)
Se2—O3—Cu2	124.02 (17)	O2^iii^—Na1—O7^vi^	99.14 (11)
Se1—O4—Cu1	116.58 (15)	O7—Na1—O6^vii^	102.55 (12)
Se1—O4—Na1^iv^	98.94 (14)	O5^v^—Na1—O6^vii^	73.44 (11)
Cu1—O4—Na1^iv^	104.54 (13)	O2^vi^—Na1—O6^vii^	132.30 (12)
Cu2—O5—Na1^v^	97.49 (13)	O4^iii^—Na1—O6^vii^	62.63 (11)
Cu2—O5—H1	116 (6)	O2^iii^—Na1—O6^vii^	115.47 (12)
Na1^v^—O5—H1	94 (5)	O7^vi^—Na1—O6^vii^	144.02 (11)

Symmetry codes: (i) −*x*, −*y*, −*z*; (ii) −*x*−1, *y*−1/2, −*z*+1/2; (iii) *x*+1, *y*, *z*; (iv) *x*−1, *y*, *z*; (v) −*x*, −*y*+1, −*z*; (vi) −*x*+1, −*y*+1, −*z*; (vii) −*x*+1, −*y*, −*z*.

### Hydrogen-bond geometry (Å, °)

**Table:**

*D*—H···*A*	*D*—H	H···*A*	*D*···*A*	*D*—H···*A*
O5—H1···O4^v^	0.80 (8)	2.00 (8)	2.786 (4)	168 (7)
O5—H2···O3^viii^	0.87 (8)	1.88 (8)	2.746 (4)	174 (7)
O6—H3···O8^ix^	0.87 (8)	1.91 (8)	2.758 (5)	163 (7)
O6—H4···O1^ii^	0.89 (8)	1.76 (8)	2.641 (4)	169 (8)

Symmetry codes: (v) −*x*, −*y*+1, −*z*; (viii) −*x*−1, *y*+1/2, −*z*+1/2; (ix) −*x*, *y*−1/2, −*z*+1/2; (ii) −*x*−1, *y*−1/2, −*z*+1/2.
